# Phase I–II study of lenalidomide and alemtuzumab in refractory chronic lymphocytic leukemia (CLL): effects on T cells and immune checkpoints

**DOI:** 10.1007/s00262-016-1922-6

**Published:** 2016-11-04

**Authors:** Maria Winqvist, Fariba Mozaffari, Marzia Palma, Sandra Eketorp Sylvan, Lotta Hansson, Håkan Mellstedt, Anders Österborg, Jeanette Lundin

**Affiliations:** 1 Department of Hematology, Karolinska University Hospital Solna, 171 76 Stockholm, Sweden; 2 Department of Oncology-Pathology, Karolinska Institutet, Stockholm, Sweden

**Keywords:** Chronic lymphocytic leukemia, Lenalidomide, Alemtuzumab, T cells, Immune checkpoints

## Abstract

This phase I–II study explored safety, immunomodulatory and clinical effects of lenalidomide (weeks 1–16) and alemtuzumab (weeks 5–16) in 23 patients with refractory chronic lymphocytic leukemia. Most patients had Rai stage III/IV disease and were heavily pretreated (median 4 prior therapies), and 61% had del(17p)/del(11q). Eleven of 19 evaluable patients (58%) responded, with a median response duration of 12 months (1–29+); time to progression was short in non-responders. Lenalidomide had a narrow therapeutic dose range, 2.5 mg/day was not efficient, and maximum tolerated dose was 5 mg/day. Grade 3–4 neutropenia and thrombocytopenia occurred in 84 and 55%, 30% had febrile neutropenia, and CMV-reactivation requiring valganciclovir occurred in 30% of patients. The frequency of proliferating (Ki67^+^) CD8^+^ T cells was increased at week 4, with further increase in both the CD4^+^ and CD8^+^ subsets (*p* < 0.01 and <0.05), which was accompanied by significant upregulation of HLA-DR after addition of alemtuzumab. Antigen-experienced cells increased at week 4 as the frequency of effector memory cells increased in the CD8^+^ subset (*p* < 0.003), while effector cells decreased in both the CD8^+^ and CD4^+^ subsets (*p* < 0.0001 and *p* < 0.01). The Th1/Th2 balance was unchanged at week 4 but shifted toward a Th2 profile after combination therapy. At end of treatment, the frequency of Th17 and regulatory T cells was reduced (*p* < 0.01), naïve T cells decreased, and effector memory T cells increased (*p* < 0.05 and *p* < 0.01). Granzyme B^+^ T cells increased at 30-week follow-up (*p* < 0.05). PD-1 expression was unaffected. In conclusion, low-dose lenalidomide and alemtuzumab induced major perturbations of T cells, including increased proliferative activity and cytotoxic potential.

**Electronic supplementary material:**

The online version of this article (doi:10.1007/s00262-016-1922-6) contains supplementary material, which is available to authorized users.

## Introduction

Chronic lymphocytic leukemia (CLL) is associated with impaired immune functions resulting in increased risk of infections and secondary tumors. Patients often have hypogammaglobulinemia and abnormalities in T and NK cells. T cell dysfunctions are due to several factors, such as defective immunological synapse formation [1], impaired cell cytotoxicity [2] and imbalance in cell subsets [3, 4].

Lenalidomide is an immunomodulatory drug which has shown anti-tumor activity in patients with relapsed and/or refractory CLL, achieving an overall response rate (ORR) of 32–47% [5–7]. The most prominent side effects of lenalidomide in CLL are neutropenia and tumor flare reaction (TFR), with a dose-dependent risk of tumor lysis syndrome [6].

The mechanisms of action for lenalidomide in CLL are not entirely understood, but might include a direct tumor cell killing effect, immune modulation and alteration of the tumor microenvironment. Altered cytokine levels, upregulation of co-stimulatory molecules on CLL cells, normalized balance of T cell subsets and improved NK and T cell function have been reported in CLL patients treated with lenalidomide (*reviewed in* [8]). Moreover, immunomodulatory drugs reduced the number of regulatory T cells (T_regs_) [9, 10], which are usually increased in patients with advanced-stage CLL [4, 9, 11] and expanded pro-inflammatory type 17 T helper (Th17) cells [10]. Furthermore, immunomodulatory drugs induced T cell activation, proliferation and cytokine production in vitro without mitogenic activity [12, 13], but by a co-stimulatory effect [13, 14]. In vitro studies have also shown that lenalidomide may improve T cell functions by repairing the defective immunological synapse formation with CLL cells [15]. In vivo, lenalidomide treatment increased the number of CD8^+^CD69^+^ cells as well as IFN-γ-producing CD8^+^ cells, indicating an enhanced cytotoxic activity [10, 16]. Lenalidomide also induced production of IL-2 and IFN-γ in vivo, mediating a shift toward a type 1 T helper (Th1) cells profile [17].

T cell activation through the TCR is regulated by a number of co-stimulatory and inhibitory signals, including immune checkpoint receptors. This ultimately controls T cell activation in lymph nodes and effector responses in peripheral tissues. In particular, the immune checkpoint receptor PD-1 is induced on activated T cells, and once bound to the ligands, PD-L1 or PD-L2, expressed on tumor cells or cells in the tumor microenvironment, reduces TCR signaling [18].

T cells in CLL patients displayed a high PD-1 expression [2, 4, 19], and chemotherapy seemed to increase the proportion of CD4^+^PD-1^+^. This increase could be reversed by lenalidomide treatment [20]. Regarding the expression of the ligand PD-L1 on CLL cells, results are conflicting [4, 15, 19]. CTLA-4 is another immune checkpoint molecule transiently expressed on activated T cells [21] inhibiting T cell activation. It has been reported that lenalidomide could overcome the inhibitory effect of CTLA-4 on T cell responses against the Epstein–Barr virus in vitro [14]. The expression profile of CTLA-4 on T cells from CLL patients has shown varying results [2, 4, 22].

Alemtuzumab is a humanized CD52 mAb that induced a response rate of 30–40% in relapsed/refractory CLL patients [23, 24]. Even though no longer approved for CLL but re-launched in multiple sclerosis, it is available through a free access program for CLL and other patients with an unmet need. Antibody-dependent cellular cytotoxicity, mainly induced by NK cells, is supposed to be an important effector function of alemtuzumab, but also depleting immune cells leading to an increased risk of opportunistic and other infections.

The rationale for combining lenalidomide with alemtuzumab in the present trial was based on the assumption that lenalidomide might potentiate the antitumor activity of alemtuzumab by stimulating NK cell-mediated antibody-dependent cellular cytotoxicity and activate T cells to counteract the alemtuzumab-induced negative effect on T cells. Moreover, a synergistic effect might be expected, as the two drugs may have preferential effects in different disease compartments, i.e., alemtuzumab mostly on bone marrow and peripheral blood and lenalidomide mainly on lymph nodes, even though late responses on lenalidomide may occur in the bone marrow.

Results of a phase I study, in which 12 refractory CLL patients were included, have previously been reported [25]. In the present report, the complete analysis of totally 23 patients is described, with a focus on analysis of changes in T cell subsets, including immune checkpoints as well as cell activation, proliferation and cytotoxic markers.

## Materials and methods

### Study population and eligibility criteria

The study was conducted according to the Declaration of Helsinki. Informed consent was obtained from all individual participants included in the study. The study was registered at clinical trials.gov and was approved by the Swedish Medical Products Agency (EudraCT number 2007-007434-20) and the regional ethics committee. Patients with chemotherapy-refractory CLL or judged ineligible for chemotherapy due to, for example, del(17p)/*TP53* mutation or severe cytopenia were included in the study. The following criteria should be fulfilled as well: neutrophils ≥0.5 × 10^9^/L, platelets ≥25 × 10^9^/L, creatinine ≤177 µmol/L, total bilirubin ≤26 µmol/L and Eastern Cooperative Oncology Group (ECOG) performance status ≤2.

### Treatment schedule

Lenalidomide was given continuously as a single agent for weeks 1–4 at a starting dose of 2.5 mg/day in patients 1–6 and 5 mg/day in patients 7–23 [25]. Alemtuzumab was added at week 5 (30 mg s.c. three times a week), and lenalidomide was then escalated stepwise (5–10–15 mg/day) depending on tolerability. Maximum treatment period was 16 weeks of lenalidomide and 12 weeks of alemtuzumab.

Prophylaxis for tumor lysis syndrome and TFR was provided by proper hydration, allopurinol given 3 days before start of lenalidomide, as well as 10 mg of prednisone/day during the first week of treatment and at dose escalations. All patients received infection prophylaxis with cotrimoxazole, valaciclovir and G-CSF (300 µg s.c. three times a week to normal granulocyte count). CMV monitoring was not performed, although CMV-PCR test was carried out in case of unexplained fever.

### Assessments

Response evaluation was based on the 2008 International Workshop on Chronic Lymphocytic Leukemia (iwCLL) criteria [26]. Patients were evaluated with computed tomography scan at the end of treatment as well as bone marrow biopsies at week 12 and at the end of treatment. Treatment toxicity was evaluated using National Cancer Institute (NCI) Common Terminology Criteria for Adverse Events v3.0 except for anemia, thrombocytopenia and neutropenia which were graded according to the grading scale for hematological toxicity in CLL studies [26]. Maximum tolerated dose (MTD) was defined as the dose that caused dose-limiting toxicity in less than one-third of the patients.

### Flow cytometric analysis of whole blood lymphocytes

After lysis of red blood cells, cells were washed, resuspended in BD FACSFlow (BD Biosciences, San Diego, CA, USA) and stained according to the manufacturer’s recommendation using fluorochrome-coupled antibodies for CD19, CD3, CD4, CD8, CD16, CD56, CD52, CD45RA, human leukocyte antigen–antigen D related (HLA-DR) and C-C motif chemokine receptor (CCR) 7 (Supplementary Table 1). After incubation and washing, cells were resuspended in FACSFlow and analyzed by flow cytometry using a FACSCanto II flow cytometer and analyzed by FACSDiva version 6.1.3 (BD Biosciences) or Infinicyt (Cytognos S.L., Salamanca, Spain).

### Flow cytometric analysis of PBMC

PBMC were isolated from heparinized blood by density gradient centrifugation on a Ficoll-Hypaque (GE Healthcare, Uppsala, Sweden), washed twice with Dulbecco’s phosphate-buffered saline (0.9%) (Gibco, Life Technologies, Carlsbad, CA, USA). Cells were freshly used or stored in liquid nitrogen until use.

PBMC were then washed with CSB (BioLegend, San Diego, CA, USA) and stained for PD-1, CTLA-4, PD-L1, CCR6, C-X-C motif chemokine receptor (CXCR) 3, CD25, human leukocyte antigen–antigen D related (HLA-DR) (Supplementary Table 1) and the appropriate isotype controls. After incubation for surface staining and washing, cells were resuspended in CSB and flow cytometric analysis was done by FACSCanto II flow cytometer (BD Biosciences).

For intracellular staining, cells were resuspended in fixation/permeabilization buffer, incubated for 30 min at 4 °C and washed with permeabilization buffer (eBioscience). Antibodies (FOXp3, granzyme B, perforin, Ki67) were added to the resuspended cells and incubated for 30 min at room temperature. Excess of antibodies was removed by washing twice with permeabilization buffer. The cells were then resuspended in CSB and analyzed by flow cytometry using a FACSCanto II flow cytometer and analyzed by the FACSDiva version 6.1.3 (BD Biosciences) or the FlowJo version 8.8.2 (TreeStar, Ashland, OR, USA) softwares.

### Statistical methods

Statistical analyses were performed using GraphPad Prism 6.0 and SPSS 23.0. Comparison of marker expression at different time points was done with the Wilcoxon signed-rank test for related samples. All tests were two-sided and *p* < 0.05 was considered significant. Survival analysis was done with the Kaplan–Meier method.

## Results

### Patients

Clinical characteristics of the patients are shown in Table 1. Median age was 69 years (range 61–85); 70% had Rai stage III/IV, 48% bulky disease (lymphadenopathy >5 cm), 61% 17p and/or 11q deletion and 73% an unmutated Ig heavy chain variable (IgHV) gene. Patients were heavily pretreated having received a median of 4 prior therapies (range 1–7). Three patients had previous allo-SCT.

**Table 1:** Patient characteristics (*n* = 23)

	Median (range)	%
Age (years)	69 (61–85)	
Gender		
Male		61
Female		39
Number of previous regimens	4 (1–7)	
ECOG		
0		43
1		48
2		9
Rai stage		
I		26
II		4
III		26
IV		44
Cytogenetic abnormality		
17p- or *TP53* mutation		43
11q-		30
13q-		52
12+		13
Normal		17
Lymph nodes >5 cm		48
Unmutated IgHV gene		73
Refractory to fludarabine or bendamustine		56

### Dose and MTD

The first six patients received a starting dose of 2.5 mg lenalidomide/day. Four of these patients progressed early, and the dose was considered ineffective in advanced-phase progressive CLL [25]. After an amendment, the subsequent 17 patients received a starting dose of 5 mg/day. Median length of lenalidomide therapy was 12 weeks (range 0–16) and for alemtuzumab 10 weeks (range 0–12). Eleven patients (48%) completed all 16 weeks of lenalidomide and the 12 weeks of alemtuzumab therapy. MTD of lenalidomide, in combination with alemtuzumab, was 5 mg/day. Only two patients tolerated a lenalidomide dose of 10 mg/day for more than 4 weeks, and no patient reached a dose of 15 mg/day. The most common dose-limiting toxicity was grade 3–4 neutropenia (15/17 of patients).

### Efficacy

Four patients discontinued lenalidomide within 8 weeks due to causes other than disease progression. In one patient, the drug was withdrawn after two doses due to rapidly deteriorating general condition. Three patients stopped treatment after 3, 4 and 7 weeks, respectively, for various medical reasons (exfoliative dermatitis, legionella pneumonia and other malignancy, respectively). The ORR in the intention-to-treat population was 48% (11/23) including two complete responses and nine partial responses. When excluding the four above-mentioned patients, ORR was 58% (11/19). Responding patients received a median total lenalidomide dose of 520 mg (range 42–810 mg), and non-responders received a median total dose of 177 mg (range 50–650 mg).

Three patients were excluded from the analysis of progression-free survival, response duration and time to next treatment since after study completion they received either donor lymphocyte infusion (two patients) or allo-SCT (one patient). Median progression-free survival was 6 months (range 0–37+ months) (Supplementary Fig. 1) with five patients having a progression-free survival exceeding 12 months. Median overall survival (OS) was 17 months (0–65+) (Supplementary Fig. 1). All but one of the surviving patients are currently on treatment with ibrutinib or idelalisib.

Median response duration time of the eight responding patients was 12 months (range: 1–29+ months) (Supplementary Fig. 1) and median time to treatment failure (next treatment or death) 18 months (range 3–52+ months).

### Toxicity

The most common grade 3–4 adverse events were hematological: neutropenia (84%) and thrombocytopenia (55%). No patient developed anemia ≥grade 3. The most common non-hematological grade 3–4 adverse events were febrile neutropenia (*n* = 7), pneumonia (*n* = 5), CMV-reactivation (*n* = 2), thrombosis (*n* = 2) and fever of unknown origin (*n* = 2). CMV-reactivation requiring valganciclovir therapy occurred in seven patients (30%).

Seven patients (30%) developed transient TFR (grade I–II). Six of these responded to treatment, including five partial responses and one complete response. The only patient with TFR who did not respond discontinued treatment early due to legionella pneumonia.

### Immune responses

#### T cells, NK cells and Natural killer T (NKT) cells were markedly reduced by the combination treatment

**Fig. 1:**
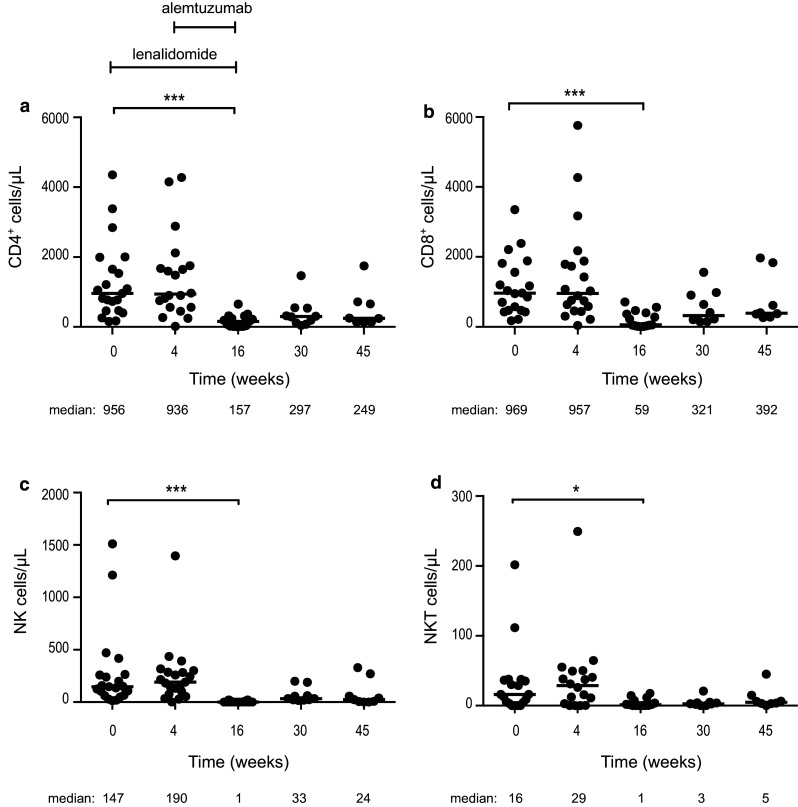
Absolute numbers of **a** CD4^+^, **b** CD8^+^, **c** NK and **d** NKT cells at baseline, during and after therapy. Lenalidomide was administered on weeks 1–16 and alemtuzumab weeks 5–16. Median number of cells/µL at each time point is depicted under each figure. * *p* < 0.05, ** *p* < 0.01, *** *p* < 0.001

After 4 weeks of single-agent lenalidomide treatment, no statistically significant changes were observed in the numbers of CD4^+^ T cells, CD8^+^ T cells, NK cells and NKT cells. After adding alemtuzumab, all the lymphoid subsets decreased dramatically at week 16 and remained low in most patients at follow-up (weeks 30 and 45) (Fig. 1). The median numbers of CD4^+^ and CD8^+^ T cells were 157 and 59 cells/µL at week 16 (end of treatment), 297 and 321 cells/µL at week 30, and 249 and 392 cells/µL at week 45, respectively (Fig. 1a, b).

The proportion of CD52^−^ T cells increased during treatment. At baseline, the number of CD52^−^CD3^+^ cells was low (median 18 cells/µL), whereas the number of CD52^+^CD3^+^ cells was high (median 1828 cells/µL). At the end of treatment, the frequency was reversed with CD52^−^ cells comprising more than 85% of all T cells. Also, total CD52^−^ T cell numbers increased during treatment (median 292, 544 and 343 cells/µL at weeks 16, 30 and 45, respectively) (data not shown).

#### Activated (HLA-DR^+^) and proliferating (Ki67^+^) T cells increased during lenalidomide treatment

**Fig. 2:**
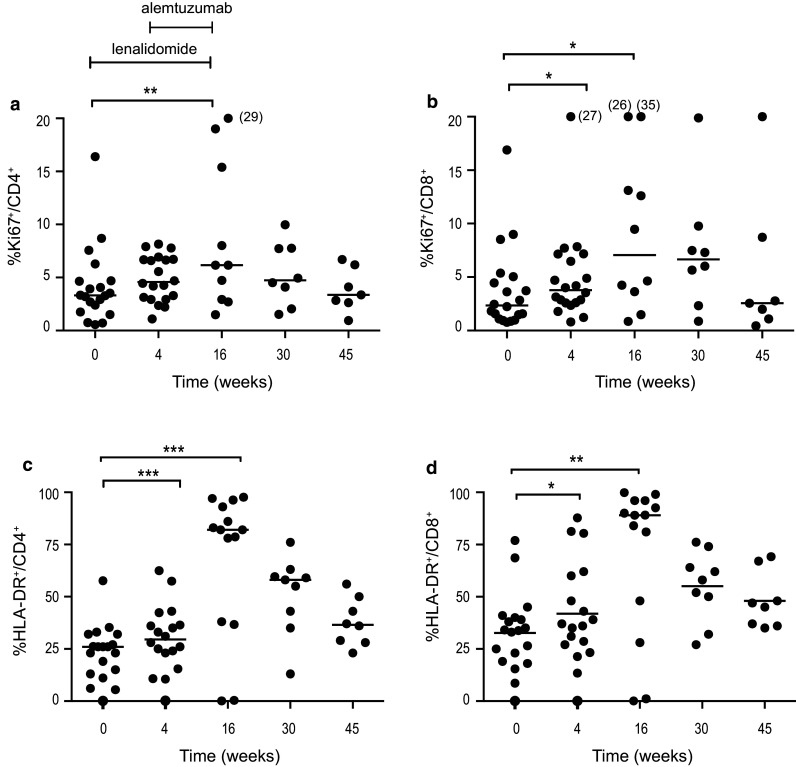
Percentage of **a** Ki67^+^CD4^+^, **b** Ki67^+^CD8^+^, **c** HLA-DR^+^CD4^+^ and **d** HLA-DR^+^CD8^+^ cells at baseline, during and after therapy. Lenalidomide was administered on weeks 1–16 and alemtuzumab weeks 5–16. * *p* < 0.05, ** *p* < 0.01, *** *p* < 0.001

After 4 weeks of lenalidomide treatment, no significant increase was observed in the frequency of Ki67^+^CD4^+^ cells (Fig. 2a), while the frequency of Ki67^+^CD8^+^ cells increased from 2.3% (median) (range 0.8–16.9) at baseline to 3.8% (range 0.8–27.0) at week 4 (*p* < 0.05) (Fig. 2b). At the end of treatment (week 16), Ki67^+^ cells among both the CD4^+^ and CD8^+^ subsets had further increased (median 6.2 and 7.0%, respectively, *p* < 0.006 and *p* < 0.05) (Fig. 2a, b), and then, normalization gradually occurred. As preliminarily reported earlier [25], a significant increase was observed in the frequency of activated (HLA-DR^+^) T cells after single-agent lenalidomide treatment. The frequency increased further during the combination therapy, and HLA-DR^+^ cells increased from 25% at baseline to 82% at week 16 within the CD4^+^ subset (*p* = 0.0009) and from 33 to 89% in the CD8^+^ subset (*p* = 0.0017) (Fig. 2c, d). Similar to Ki67^+^ cells, HLA-DR^+^ expression gradually normalized during the non-treatment follow-up period. There was no significant change in the proportion of T cells expressing perforin/granzyme B during treatment with lenalidomide alone. The combination treatment significantly increased the proportion of granzyme B^+^ cells within CD4^+^ (*p* < 0.05) as well as CD8^+^ T cells (*p* < 0.05) from baseline until week 30 of follow-up (data not shown).

#### A shift toward a type 2 T helper (Th2) cells profile and reduced frequency of Th17 and T_regs_ were observed during combination therapy

**Fig. 3:**
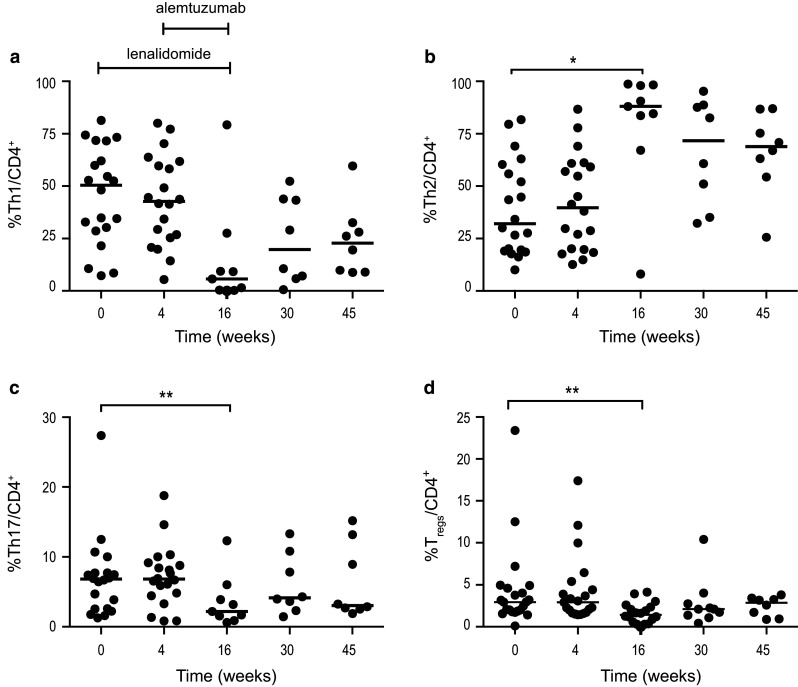
Percentage of **a** Th1, **b** Th2, **c** Th17 and **d** T_reg_ cells at baseline, during and after therapy. Lenalidomide was administered on weeks 1–16 and alemtuzumab weeks 5–16. * *p* < 0.05, ** *p* < 0.01, *** *p* < 0.001

T helper subpopulations were defined by CCR6 and CXCR3 expression as Th1 (CCR6^−^/CXCR3^+^), Th2 (CCR6^−^/CXCR3^−^) and Th17 (CCR6^+^/CXCR3^−^) cells. T_regs_ were defined as CD4^+^, CD25^high^ and FOXp3^+^. The Th1/Th2 balance (Fig. 3) was unaffected by lenalidomide therapy alone (weeks 1–4), but then the frequency of Th1 cells decreased from 50% (median) (range 7–81%) at baseline to 6% (range 0–79%) at week 16 (not significant) (Fig. 3a). The proportion of Th2 cells increased from 32% (median) (10–82%) at baseline to 88% (8–99%) at the end of the combination therapy (*p* < 0.05). The proportion of Th2 cells remained high during follow-up (Fig. 3b). The median percentage of Th17 cells decreased significantly during combination therapy from 7% at baseline to 2.2% at week 16 (*p* = 0.004) (Fig. 3c). The decrease in T_regs_ observed in part I of the study [25] was confirmed in the present extended analysis (*p* = 0.003) (Fig. 3d).

#### A shift in memory T cell subsets was observed during therapy

**Fig. 4:**
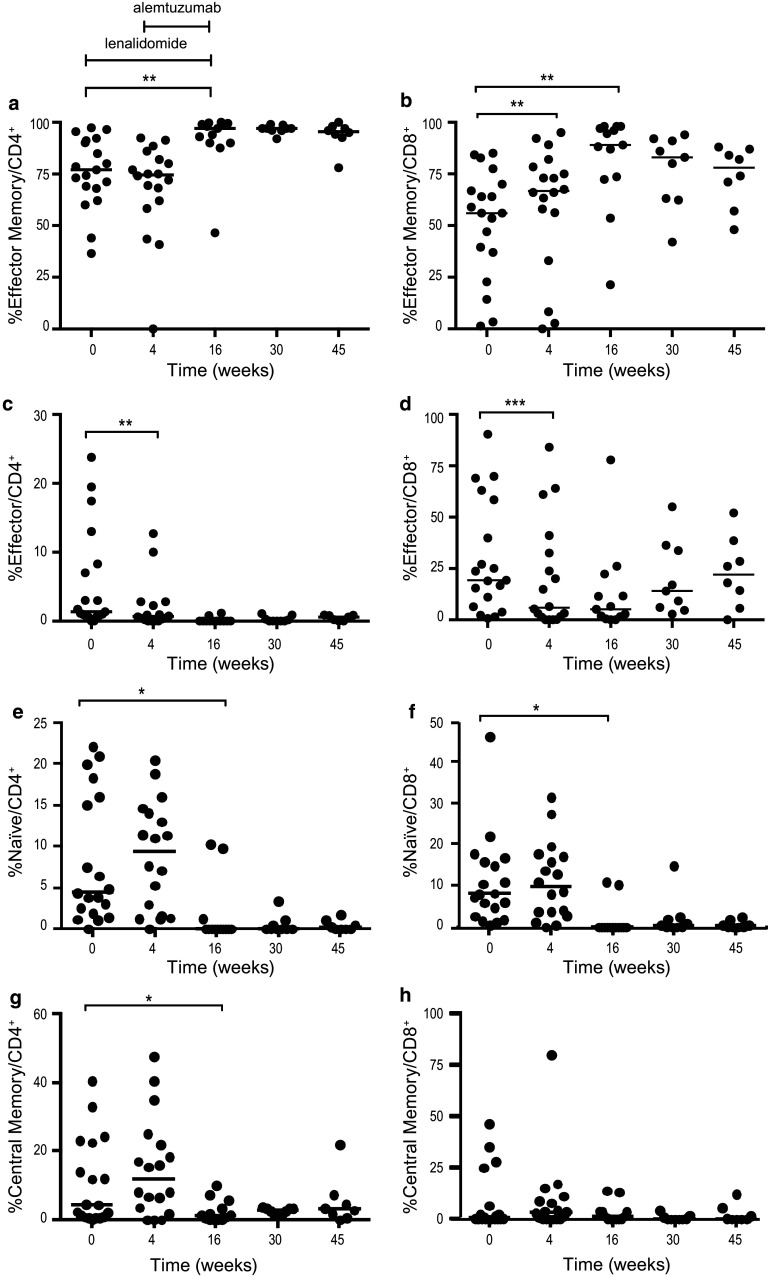
Percentage of T cell subpopulations **a** effector memory CD4^+^, **b** effector memory CD8^+^, **c** effector CD4^+^, **d** effector CD8^+^, **e** naïve CD4^+^, **f** naïve CD8^+^, **g** central memory CD4^+^, **h** central memory CD8^+^ at baseline, during and after therapy. Lenalidomide was administered on weeks 1–16 and alemtuzumab weeks 5–16. * *p* < 0.05, ** *p* < 0.01, *** *p* < 0.001

CD4^+^ and CD8^+^ memory cell subsets were identified by CCR7 and CD45RA expression. The percentages of naïve (CD45RA^+^/CCR7^+^), central memory (CD45RA^−^/CCR7^+^), effector memory (CD45RA^−^/CCR7^−^) and effector (CD45RA^+^/CCR7^−^) cells were determined. The results for effector memory cells are shown in Fig. 4a and b. After 4 weeks of lenalidomide treatment, the percentage of effector memory CD8^+^ cells increased (*p* < 0.003) (Fig. 4b), while the percentage of effector cells (CD4^+^ and CD8^+^) decreased (*p* < 0.01 and *p* < 0.0001) (Fig. 4c, d). At the end of the combination treatment (week 16), the percentages of naïve CD4^+^ and CD8^+^ T cells decreased (*p* < 0.05) (Fig. 4e, f). The percentage of effector memory cells increased significantly within both CD4^+^ and CD8^+^ cells (*p* < 0.01) (Fig. 4a, b). Central memory T cells appeared to be less affected (Fig. 4g, h).

#### PD-1 expression remained unaffected by treatment

**Fig. 5:**
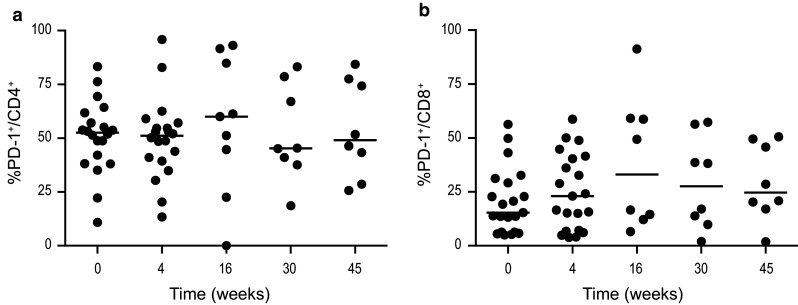
Percentage of **a** PD-1^+^CD4^+^ and **b** PD-1^+^CD8^+^ cells at baseline, during and after therapy. Lenalidomide was administered weeks on 1–16 and alemtuzumab weeks 5–16. * *p* < 0.05, ** *p* < 0.01, *** *p* < 0.001

The percentage of PD-1^+^ cells before treatment was 53% (median) in the CD4^+^ cells and 15% (median) in the CD8^+^ cells. The proportion of PD-1-expressing cells remained unchanged in both CD4^+^ (Fig. 5a) and CD8^+^ (Fig. 5b) T cells during lenalidomide, combination therapy and follow-up. No expression of PD-L1 was detected on freshly isolated CLL cells at study start or during therapy. Neither was CTLA-4 surface expression on T cells observed at study inclusion nor throughout therapy.

## Discussion

Until recently, patients with high-risk CLL [refractory/relapsed or with del(17p)/*TP53* mutation] had a very poor prognosis [27, 28]. New drugs, such as ibrutinib, venetoclax and idelalisib, which were not available for CLL patients at the initiation of this study, are now changing the landscape of CLL treatment proving to be highly effective in relapsed or refractory patients even with high-risk cytogenetics [29–32]. Despite the impressive clinical results obtained by, e.g., ibrutinib, patients with del(17p)/*TP53* mutation, still relapse and have a relatively short OS [33, 34]. Patients who relapse after treatment with the new drugs or do not tolerate them may still need the previous generation of anti-CLL agents such as alemtuzumab (currently available through a free access program) or other active agents such as lenalidomide.

Lenalidomide as a single agent has shown to be active in CLL [5–7]. It is explored as maintenance therapy for CLL in an ongoing phase III trial and in combination with other agents in phase II studies [35–40].

We report here the final results of a phase I–II trial exploring safety and clinical efficacy of lenalidomide in combination with alemtuzumab in advanced-stage CLL, with a focus on the effects of the two drugs on T cell subsets.

As for part I of the study [25], the present full study report confirmed an acceptable safety profile of the combination. However, grade 3–4 infections appeared to be more frequent than that reported in previous studies in which each drug was used separately [6, 23, 24]. The incidence of symptomatic CMV-reactivation (30%) was similar to that reported with alemtuzumab alone. Thus, the immune-enhancing potential of lenalidomide seemed not to translate into a reduced risk of infections. Higher doses of lenalidomide might have been more effective, but dose escalation was hindered by the low MTD of only 5 mg/day. Notably, grade 3–4 neutropenia occurred in more than 80% of patients, despite the use of G-CSF prophylaxis. This is in contrast to most previous studies reporting a tolerated dose of 10 mg [8, 41], even though one study noted that lenalidomide in heavily pretreated patients was feasible only at a dose of 5 mg [42]. Despite the low dose of lenalidomide, ORR of our drug combination appeared to be clinically meaningful. More than half of our elderly poor-prognosis patients with advanced-stage CLL responded and those who tolerated therapy reached a reasonably long response duration time. However, the results of the present study should be interpreted with caution as the number of patients was low.

Effects of lenalidomide indicated that immune stimulatory effects on T cells started early in spite of the low dose of lenalidomide. Not only activation (HLA-DR expression) was observed, but also an increased proportion of Ki67^+^CD8^+^ T cells at week 4. This was accompanied by a relative increase in CD8^+^ effector memory cells and a decrease in CD8^+^ effector cells, indicating an increase in antigen-experienced (CD45RA^−^) CD8^+^ cells which is in line with what have been observed in multiple myeloma patients [43, 44].

A limitation of our study is the marked T cell-depleting effect of alemtuzumab, which complicated the analysis of total T cell subsets. We therefore focused on the relative changes within major subsets, as the functions of remaining T cells in blood after alemtuzumab therapy are likely to be of importance for the immune status of the patients. As described previously [45], most post-treatment T cells were CD52^−^. The total CD4^+^ T cell numbers at the end of the combination treatment are slightly higher in the current study than after treatment with alemtuzumab alone [23].

At week 16, i.e., at the end of treatment, an increase in the percentage of Th2 cells was noted accompanied by a decrease in T_reg_, Th17 and naïve T cells. This is in contrast to previous results, suggesting that lenalidomide could cause a shift from Th2 to Th1 cells [17]. Our observations are most likely attributed to alemtuzumab therapy, similar to the relative increase in Th2 cells during alemtuzumab treatment seen in patients with multiple sclerosis [46]. Furthermore, an increase in the proportion of granzyme B^+^ T cells was noted, implying an enhanced cytotoxic potential.

PD-1 binds through its ligands on immune cells and target cells countering TCR-induced T cell proliferation, cytotoxicity and cytokine production [18]. Studies in CLL have suggested that lenalidomide might affect the expression of the PD-1/PD-L1 receptors [15, 20]. However, we were not able to confirm those findings, which is in line with a study in multiple myeloma patients where no difference in the frequency of CD8^+^PD1^+^ cells could be noted comparing untreated and lenalidomide-treated patients [43].

Moreover, we did not find PD-L1 expression on CLL cells, which is in contrast to previous studies [15, 19]. However, preliminary results from a phase II study applying PD-1 blockade in relapsed/refractory CLL, including patients with Richter’s transformation, showed promising clinical activity on the transformed cells, but not against the untransformed CLL clone [47], which might support the absence of PD-L1 on the CLL clone.

In conclusion, our study showed that lenalidomide alone or in combination with alemtuzumab induced various immunomodulatory effects on T cells in CLL patients. Further studies on lenalidomide as an immune-enhancing agent in CLL and other disorders are warranted.

### Electronic supplementary material

Below is the link to the electronic supplementary material.

Supplementary material 1 (PDF 671 kb)

## Abbreviations

CCR: C-C motif chemokine receptor
CLL: Chronic lymphocytic leukemia
CXCR: C-X-C motif chemokine receptor
ECOG: Eastern Cooperative Oncology Group
HLA-DR: Human leukocyte antigen–antigen D related
IgHV: Ig heavy chain variable
MTD: Maximum tolerated dose
NKT cell: Natural killer T cell
ORR: Overall response rate
TFR: Tumor flare reaction
Th1 cell: Type 1 T helper cell
Th2 cell: Type 2 T helper cell
Th17 cell: Type 17 T helper cell
T_reg_: Regulatory T cell
