# Supplementary material for: Phase I–II study of lenalidomide and alemtuzumab in refractory chronic lymphocytic leukemia (CLL): effects on T cells and immune checkpoints
Source: Cancer Immunol Immunother. 2016 Nov 4;66(1):91–102. doi: 10.1007/s00262-016-1922-6 (PMC5222940; doi:10.1007/s00262-016-1922-6)
Supplement: Supplementary file 1 — Supplementary material 1 (PDF 671 kb) [file 262_2016_1922_MOESM1_ESM.pdf]

Supplementary Figure 1. Response duration, PFS and OS for patients treated with lenalidomide and alemtuzumab

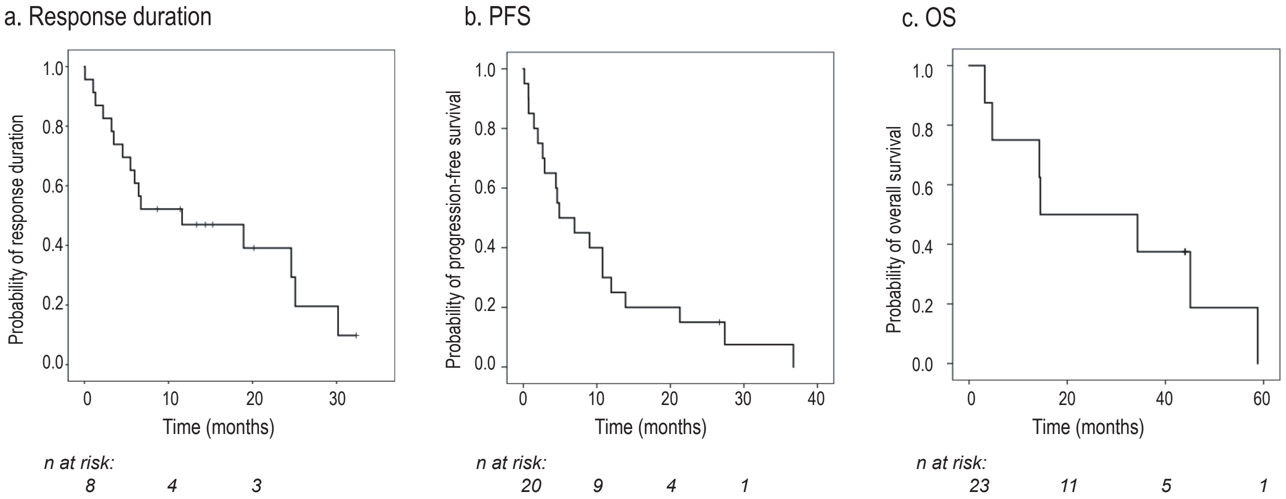

Supplementary Table 1

Cell marker	Fluorochrome	Manufacturer
CD19	PeCy7<br>APC-H7<br>Pacific Blue	BD Biosciences, SanDiego, CA, USA<br>BD Biosciences<br>BioLegend, San Diego, CA, USA
CD3	PeCy7<br>APC<br>V450	BD Biosciences<br>BD Biosciences<br>PharMingen, San Diego, CA, USA
CD4	PeCy7<br>APC-H7<br>FITC	BD Biosciences<br>BD Biosciences<br>eBioscience, San Diego, CA, USA
CD8	PerCP<br>APC<br>Pacific Blue<br>V500 Horizon	BD Biosciences<br>BD Biosciences<br>PharMingen<br>BD Biosciences
CD16	Pacific Blue	PharMingen
CD56	Pe<br>PeCy7	BD Biosciences<br>BD Biosciences
CD52	FITC	Caltag, Buckingham, UK
CD45RA	Pacific Blue	BioLegend
CCR7	Pe	R&D Systems, Minneapolis, MN, USA
PD-1 (CD279)	Pe	BD Biosciences
CTLA-4 (CD152)	Pe	BD Biosciences
PD-L1 (CD274)	Pe	BD Biosciences
CCR6 (CD196)	Pe	BD Biosciences
HLA-DR	PerCP<br>APC-Cy7	BioLegend<br>BioLegend
CXCR3 (CD183)	APC	BD Biosciences
CD25	APC	eBioscience
FOXp3	Pe	eBioscience
Ki67	AF647	BioLegend
Perforin	FITC	BD Biosciences
Granzyme B	FITC	BD Biosciences
